# Effect of Post-Translational Amidation on Islet Amyloid Polypeptide Conformational Ensemble: Implications for Its Aggregation Early Steps

**DOI:** 10.3390/ijms17111896

**Published:** 2016-11-14

**Authors:** Linh Tran, Tâp Ha-Duong

**Affiliations:** BioCIS, Université Paris-Sud, CNRS, Université Paris-Saclay, 92290 Châtenay-Malabry, France; thi-thuy-linh.tran@u-psud.fr

**Keywords:** IAPP, intrinsically disordered protein, transient secondary structures, replica exchange molecular dynamics, conformational selection mechanism

## Abstract

The human islet amyloid polypeptide (hIAPP) is an intrinsically disordered protein that can self-assemble into fibrillar aggregates that play a key role in the pathogenesis of the type II diabetes mellitus. hIAPP can transiently adopt α-helix and β-strand conformations that could be important intermediate species on the fibrillization pathway. However, experimental studies of the monomeric peptide conformations are limited due to its high aggregation propensity, and the early steps of the hIAPP association are not clearly characterized. In particular, the question of whether the aggregation-prone conformation is α-helical or β-strand-rich is still debated. In this study, combining extensive all-atom molecular dynamics (MD) and replica exchange molecular dynamics (REMD) simulations in explicit water, we shed some light on the differences between the amidated and non-amidated hIAPP conformational ensembles. Our study shows that, when compared to the amidated monomer, the non-amidation of hIAPP induces a significantly lower propensity to form β-strands, especially aggregation-prone β-hairpins. Since the fibrillization of the non-amidated hIAPP is significantly slower than that of the amidated peptide, this indicates that the early steps of the peptide oligomerization involve the association of β-hairpins or β-strands structures.

## 1. Introduction

Cosecreted with the insulin by the islet β-cells in the pancreas, the human islet amyloid polypeptide (hIAPP) is a normally soluble hormone that plays a role in regulating the blood glucose level. In particular, hIAPP modulates the rate of insulin-stimulated glucose uptake by inhibiting the synthesis of glycogen in skeletal muscle [1,2]. Like an increasing number of proteins [3,4], hIAPP can self-aggregate into oligomers and insoluble amyloid fibrils that are found in the pancreatic tissues of most of type II diabetes patients [5,6,7]. Although the question is still debated, the early steps of the fibrillization process seem to be critical for the dysfunction and/or the death of pancreatic β-cells [8,9,10]. However, despite various extensive studies, the detailed mechanism of hIAPP aggregation and the structure of the early oligomers are not clearly characterized yet [11]. In particular, different studies indicated different intermolecular interfaces in the low order oligomers [12], including helix–helix interactions [13,14,15] and β-strand associations [16,17,18].

The 37 amino acid sequence of hIAPP is KCNTATCATQ${}^{10}$ RLANFLVHSS${}^{20}$ NNFGAILSST${}^{30}$ NVGSNTY${}^{37}$-NH${}_{2}$. It should be noted that, after maturation process and post-translational modifications, the physiologically relevant form of hIAPP possesses a disulfide bond between Cys2 and Cys7 and an amidated C-terminus, which are both required for normal biological activity [2]. Nevertheless, to study the detailed mechanism of hIAPP aggregation in vitro, large quantities of pure materials are required and have to be produced by chemical synthesis or recombinant DNA techniques. In the latter approach, the hIAPP post-translational modifications are difficult to be controlled, and recombinant hIAPP is non-amidated at its C-terminal end. Interestingly, the non-amidated hIAPP is still amyloidogenic and cytotoxic [19], but its kinetics and mechanism of oligomerization differ from those of the amidated peptide [20,21,22,23,24]. In particular, several studies demonstrated that the fibrillization of non-amidated hIAPP is significantly slower than the amidated one [22,23,24]. These studies offer an opportunity to better understand the early events of the hIAPP aggregation by comparing the conformational dynamics of the amidated and non-amidated peptides.

In the presence of sodium dodecyl sulfate (SDS) micelles, the three-dimensional structure of the non-amidated and amidated hIAPP monomers are quite comparable. Indeed, the NMR structure of the micelle-bound non-amidated hIAPP, solved at pH 4.6 by Patil et al. (PDB ID: 2KB8), exhibits a very stable α-helix at region 5–16, and a less stable second one at residues 22–28 [25]. Similarly, the NMR experiments at pH 7.3 by Nanga et al. (PDB ID: 2L86) showed that the amidated peptide has an overall kinked conformation with α-helices located at residues 7–17 and 21–28 [26]. It could be noted that the last nine residues of the non-amidated peptide are unfolded [25], whereas a helical conformation can be detected at position 33–35 of the amidated hIAPP [26].

In contrast, the monomeric hIAPP three-dimensional structure is less defined in the absence of lipid-like molecules. It is widely considered as a natively unstructured peptide which can transiently adopt α-helix or β-strand conformations. Indeed, various biophysical studies, including circular dichroism (CD) and NMR experiments, indicated that the amidated hIAPP monomer has a significant propensity to form α-helical structures around the N-terminal region 5–20 [20,27,28,29,30]. However, large β-strand structures were also detected by ion-mobility mass spectrometry experiments [31]. However, to the best of our knowledge, the impact of the C-terminal amidation upon the hIAPP monomer conformations was not yet experimentally investigated.

Alternatively, the structural dynamics of hIAPP monomer can be studied using computational approaches. Among them, molecular dynamics (MD) and replica exchange molecular dynamics (REMD) simulations are often used for their ability to explore the biomolecule conformational ensembles. We recently reported a short review of computational studies on full-length hIAPP to reveal the most converging conformational states of its monomeric and oligomeric forms in solution and in interaction with lipid membrane [32]. It was concluded that hIAPP preferentially adopts α-helical conformations at the N-terminal segment and β-strands at both the N- and C-terminal regions. However, again, the role of the C-terminus amidation in the hIAPP structural dynamics and its oligomer stability is not clearly established [22]. In order to shed some light on the impact of the hIAPP amidation on its conformations and aggregation propensity, we reported here the results of extensive all-atom MD and REMD simulations of the non-amidated peptide in solution. Our computational study shows that the non-amidation of hIAPP decreases the propensity of the monomers to form β-conformations, especially at the C-terminal region.

## 2. Results

### 2.1. MD Simulations of the Amidated and Non-Amidated hIAPP

**Figure 1:**
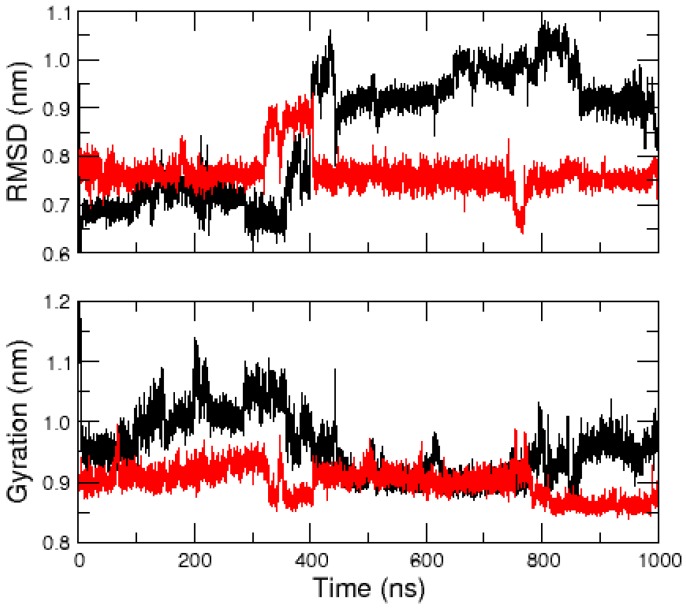
Time evolution of the RMSD relative to the 2L86 structure (**top**) and of the radius of gyration (**bottom**) for the amidated (**black** line) and non-amidated (**red** line) hIAPP monomer.

**Figure 2:**
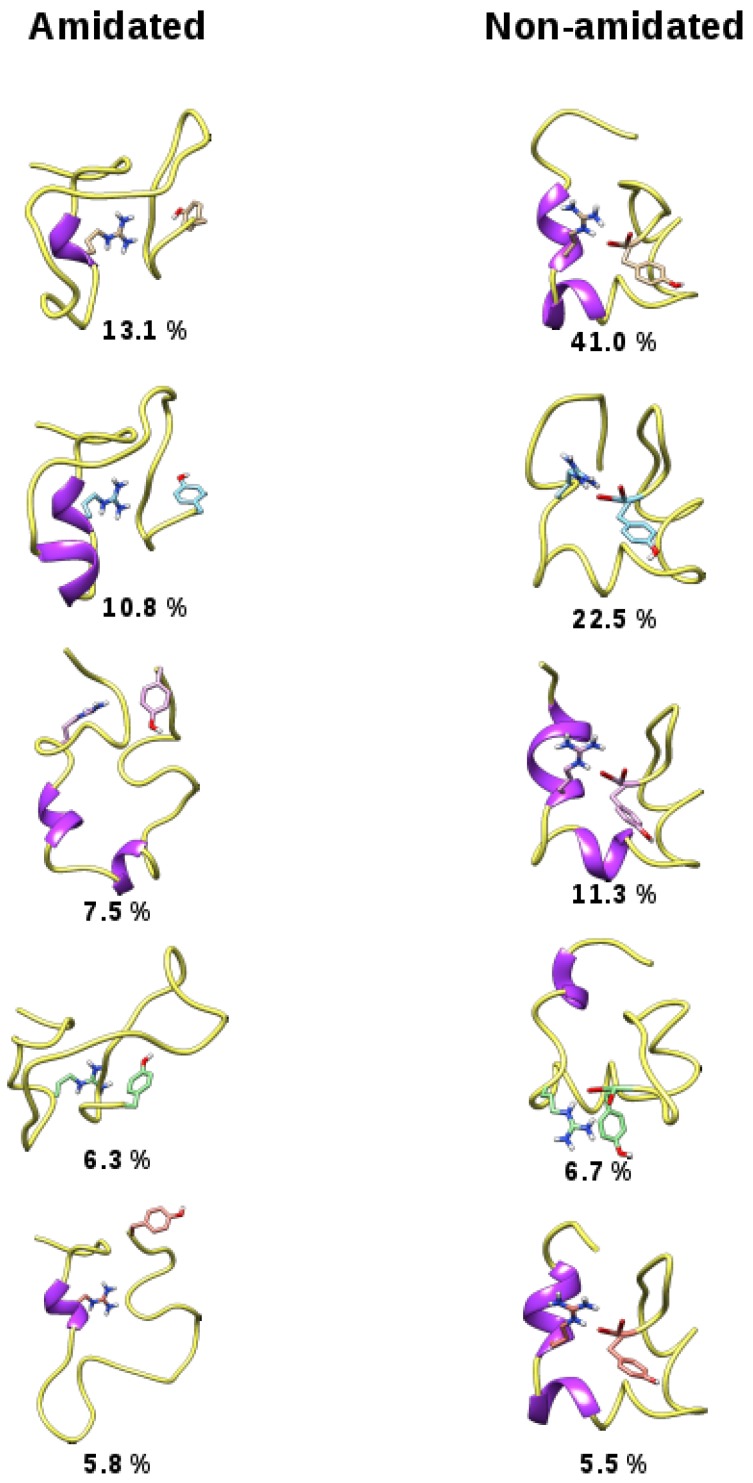
Representative structures of the five most populated clusters calculated from the 1 μs trajectory of the amidated (**left** column) and the non-amidated (**right** column) hIAPP peptide. The two residues Arg11 and Tyr37 are highlighted with stick representations.

The amidated and non-amidated hIAPP monomer structures were resolved by NMR experiments in the presence of SDS micelles, and, overall, both peptides have helical conformations [25,26]. In this section, we report the results of microsecond MD simulations to study the effect of hIAPP amidation on the unfolding and stability of these α-helices in the absence of SDS molecules. The time evolution of the peptide root mean square deviation (RMSD) with respect to the 2L86 structure and that of their radius of gyration (Figure 1) indicate that the amidated hIAPP explored more diverse and more extended conformations than the non-amidated one. The latter remained more constrained and more compact, as illustrated by Figure 2, which displays representative conformations of the five most populated clusters for both monomers.

**Figure 3:**
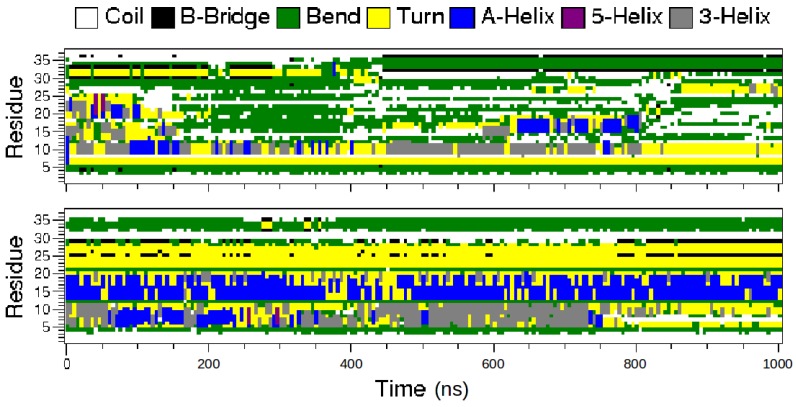
Time evolution of the secondary structures of the amidated (**top**) and non-amidated (**bottom**) hIAPP residues.

**Figure 4:**
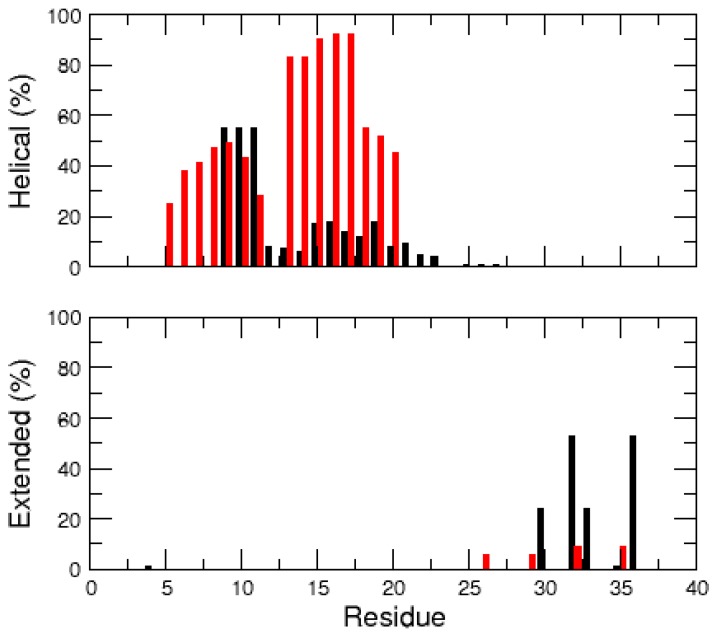
Secondary structure probability (%) of the amino acids of the amidated (**black** bar) and non-amidated (**red** bar) hIAPP monomer.

From the time evolution of the hIAPP secondary structures (Figure 3), it can be observed that the amidated peptide unfolded and rapidly lost its helices, whereas those of the non-amidated form are more stable. On average, over the 1 μs trajectories, the amidated hIAPP has 9% and 4% of its residues in α-helix and β-strand conformations, respectively. Conversely, the non-amidated peptide has significantly higher α-helix and lower β-strand contents with 25% and 2%, respectively. The secondary structure probability of the each hIAPP residue is detailed in Figure 4. In both peptide forms, the N-terminal regions 5–10, mainly keeps its helix conformation, but the segments 13–20 are clearly less helical in the amidated hIAPP than in the non-amidated one. Interestingly, the amidation of hIAPP induced the formation of stable β-conformations at the peptide C-terminal regions 30–37 when compared to the non-amidated form (Figure 3 and Figure 4). Using also a standard MD approach, Andrews and Winter similarly reported that the amidated human IAPP has a higher propensity to form β-structures than the non-amyloidogenic rat peptide [33], pointing out the importance of these β-structures for the amyloid formations.

**Figure 5:**
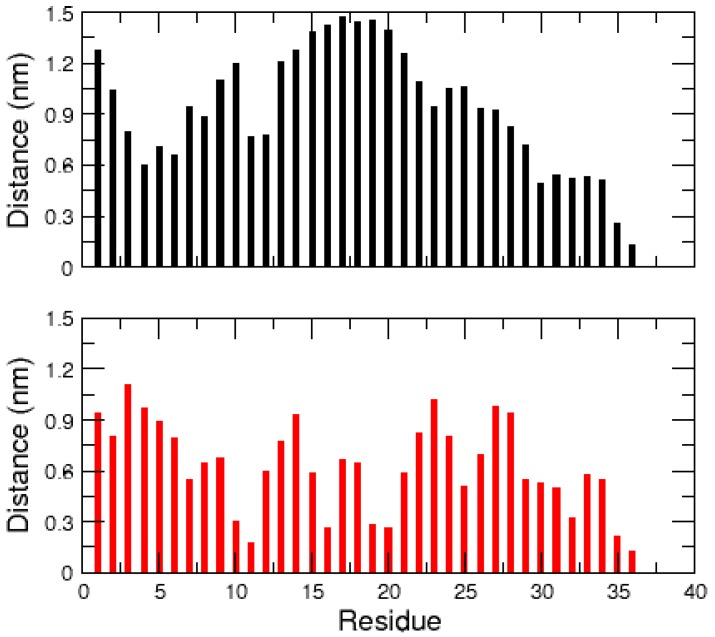
Average distances between the C-terminal residue Tyr37 and all other amino acids of the amidated (**top**) and non-amidated (**bottom**) hIAPP monomer.

In order to explain the difference between the conformational ensembles of the amidated and non-amidated hIAPP, the average distances between the C-terminal Tyr37 and all other residues were calculated over the two MD trajectories (Figure 5). In the amidated peptide, the neutral residue Tyr37 makes contact with no particular other hIAPP amino acids. In contrast, the negatively charged Tyr37 of the non-amidated hIAPP makes steady contact with residues Gln10, Arg11, Leu16, Ser19 and Ser20. This indicates that the polar interactions of the C-terminal carboxylic acid with Gln10, Ser19 and Ser20, and more especially the salt-bridge with the positively charged Arg11 (Figure 2), stabilize the compact conformation of the non-amidated hIAPP and hinder its α-helix unfolding. Conversely, the neutral C-terminus of the amidated peptide has more unrestricted movements, making easier the unfolding of its helical regions 13–20.

### 2.2. REMD Simulations of the Non-Amidated hIAPP

**Figure 6:**
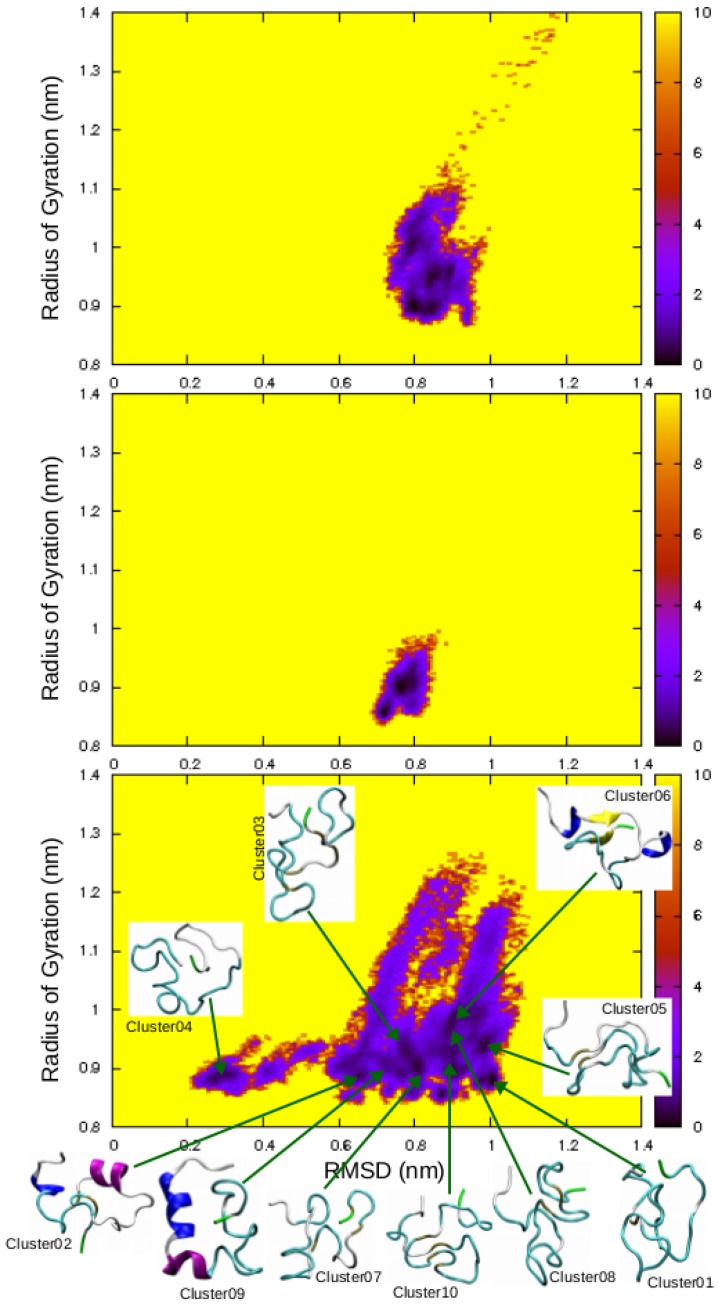
hIAPP free energy (kcal/mol) surfaces as a function of the RMSD relative to the initial random coil conformation and of its radius of gyration. (**Top**) MD simulation of the amidated hIAPP; (**Middle**) MD simulation of the non-amidated peptide; (**Bottom**) REMD simulations of the non-amidated form.

Despite an aggregation kinetics different from that of the amidated hIAPP, the non-amidated peptide can still form fibrils. Thus, it may have a significant propensity to undergo conformational transitions towards β-strand-rich structures, which were not observed in the previous MD simulation. Subsequently, we performed REMD simulations of the non-amidated hIAPP, in order to explore more exhaustively its conformational ensemble and detect these β-structures. As shown by the free energy maps displayed in Figure 6, the peptide conformational space explored by REMD is much wider than those revealed by previous MD simulations. The REMD simulations uncovered at least ten metastable conformations (represented by dark stains in the free energy maps) with RMSD relative to the initial random coil ranging from 2 to 10 Å, indicating a wide variety of structures.

To gain insight into these metastable states, the REMD sampled conformations were clustered based on their structural similarity with an RMSD threshold of 0.2 nm. Over the 560 generated clusters, the 10 most populated ones were characterized by their RMSD with respect to the initial random coil, radius of gyration, α-helix and β-strand contents (Table 1). A representative conformation of each of these 10 clusters was located on the non-amidated hIAPP free energy map (Figure 6). We observed that, in contrast to the most populated conformations explored by the classical MD simulation which are mainly helical (Figure 2), most of these REMD metastable states adopt random coil conformations without any secondary structure. Three clusters (cluster02, cluster06 and cluster09) have two α-helices at similar regions, around segments 5–11 and 13–21, and only cluster06 has a small β-sheet composed of two parallel β-strands at residues 9–10 and 34–35. Interestingly, cluster09 has a morphology similar to that of the SDS micelle-bound hIAPP (PDB ID: 2L86), indicating that the binding of hIAPP monomers to lipid membrane may occur through a conformational selection mechanism.

**Figure 7:**
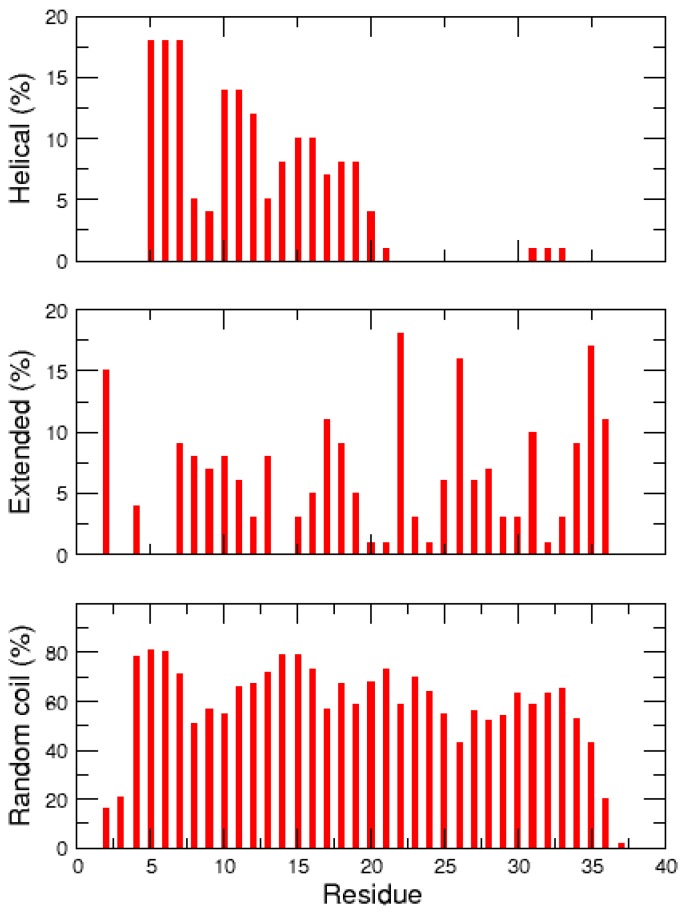
Percentage over the REMD trajectories of helical (**top**); extended (**middle**) and coil (**bottom**) secondary structures for each residue of the non-amidated hIAPP.

The propensity of the non-amidated hIAPP residues to form helical, extended and random coil conformations is detailed in Figure 7. These data support that hIAPP is mostly non-structured, with persistent helix structures in the N-terminal regions 5–20. This observation is in good agreement with NMR experiments which also detected transient α-helical states in the regions 5–19 of the rat IAPP [34]. In addition, unlike what was observed with the single MD trajectory, the REMD simulations revealed that the non-amidated hIAPP can transiently and locally form extended secondary structures, notably at segments 7–13, 15–19, 25–31 and 35–37.

## 3. Discussion

To better understand the relationship between the conformations of hIAPP monomer and its aggregation into fibrils, several theoretical studies examined the influence of mutations on the peptide structures and their fibrillization propensity. Thus, the conformational ensemble of the human IAPP monomer was frequently compared to that one of the rat IAPP which is non-amyloidogenic [31,33,35,36,37]. It was generally shown that the rat IAPP sequence is less prone to form persistent β-strands than hIAPP, which could explain its inability to form fibrils. Similarly, extensive simulations indicate that proline substitutions, such as I26P, S28P or S29P, reduce the content of β-structures with respect to wild-type hIAPP, in correlation with lower propensities to form fibrils [36,38]. In contrast, the mutation S20G found in some Asian populations significantly increases the propensity of hIAPP to form helical and β-strand structures, which could account for the accelerated rate of amyloid formation induced by this mutation [36].

**Figure 8:**
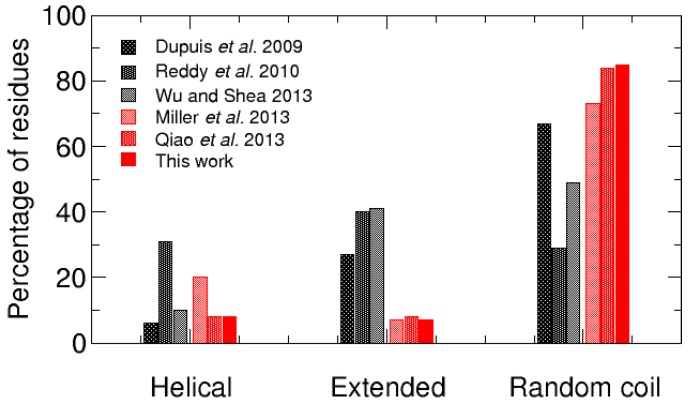
Comparison of the averaged percentage of the residues found in helical (**left**), extended (**middle**) and random coil (**right**) secondary structures for the amidated hIAPP (**grey** bars) [31,35,39] and its non-amidated form (**red** bars) [36,40].

In the same manner, the present work characterized the conformational ensemble of the non-amidated hIAPP to account for its slower rate of fibrillization than the amidated one [22,23,24]. As illustrated in Figure 6, the hIAPP conformational ensemble is explored more exhaustively with REMD than with standard MD simulations. We thus compared our results on the non-amidated peptide with other REMD data on hIAPP monomers previously reported in the literature. We found three REMD studies on the amidated peptide [31,35,39] and two other REMD simulations on the non-amidated form [36,40]. Overall, when the percentages of the hIAPP residues found in helical, extended or random coil conformation are compared (Figure 8), it is observed that the amidated and non-amidated peptides have no significantly different propensities to form α-helices (6%–31% vs. 8%–20%). In contrast, and despite the variety of the employed force fields and water models, the amidated hIAPP clearly have a higher propensity to form β-structures than the non-amidated one (27%–41% vs. 7%–8%) and, consequently, a lower percentage of residues in random coil (29%–67% vs. 73%–85%).

In both amidated and non-amidated hIAPP, the helical conformations mainly occur at the N-terminal segments 5–20, whereas the β-structures can appear at both N- and C-terminal regions of the peptide (Figure 7) [31,35,36,40]. Notably, the three REMD studies of the amidated hIAPP highlighted a significantly populated extended β-hairpin structure (with a turn around residues 20–23), which would be prone to aggregate through β-strand-β-strand interactions. This β-hairpin conformation was not retrieved in our REMD study of the non-amidated peptide, and was not observed by Miller et al. [36]. The extensive molecular simulations of the non-amidated hIAPP by Qiao et al. detected several β-hairpin structures, but their populations were very small (about 1%) compared to those of the amidated peptide (between 11% and 75%) [40]. Overall, our study emphasizes that the amidation of the hIAPP C-terminus increases the stability of β-structures, especially potential aggregation-prone β-hairpins. Our data indicate that the low population of β-strands in the non-amidated peptide is probably due to the ionic interaction between the C-terminal carboxylic acid and the Arg11 side chain, which hinder the conformational transitions from compact to extended structures.

## 4. Materials and Methods

### 4.1. Molecular Simulations

The conformational ensembles of full-length hIAPP monomers were explored using all-atom MD and REMD techniques implemented in the GROMACS-4.5.5 molecular modeling package [41,42]. Simulations were performed using the OPLS-AA force field [43] and the SPC/E water model [44]. A comparative study of different all-atom force fields suggested that OPLS-AA has the tendency to generate a good balance between α-helical and β-strand structures, whereas AMBER94 and AMBER99 over-stabilize α-helices and GROMOS96 favors β-conformation [45]. The non-bonded interactions were treated using the smooth particle mesh Ewald (PME) method [46] for the electrostatic terms and a cutoff distance of 1.2 nm for the van der Waals potentials. The covalent bond lengths were kept constant using the LINCS [47] and SETTLE [48] procedures. A leap-frog algorithm was used to integrate the equations of motion with a time step of 2 fs.

The MD initial conformation of the amidated hIAPP was taken from the SDS micelle-bound structure (PDB ID: 2L86) resolved by NMR experiments [26]. The initial non-amidated hIAPP form was derived from a similar α-helical structure. The peptides were solvated with about 4760 water molecules and their total charges were neutralized by adding sufficient sodium and chloride ions to reach the salt concentration of 0.1 mol/L. To assess their secondary structure stability, the two monomeric hIAPP systems were submitted to 1 μs MD simulations in the isothermal–isobaric (NPT) ensemble, at T=300 K and P=1 bar, using the Nose–Hoover and Parrinello–Rahman algorithms, with the time constants for coupling $\tau_{T}=0.5$ and $\tau_{P}=2.5$ ps [49,50,51].

The conformational ensemble of the non-amidated hIAPP monomer in solution was more exhaustively explored using REMD simulations [52]. For this study, the NMR structure of the SDS micelle-bound non-amidated hIAPP (PDB ID: 2KB8) [25] was first pre-equilibrated in vacuum, using a 10 ns MD simulation, to generate a random coiled conformation as the starting structure for the REMD study. In this way, the exploration of the peptide conformational space is weakly biased towards possible structured conformations. Using the same force fields and simulation parameters as previously, the REMD simulations consist in running 48 parallel MD simulations of 48 replicas of the system at different temperatures ranging from 290 K to 425 K. These temperatures were determined using the “temperature generator for REMD simulations” web server developed by Patriksson and van der Spoel [53] with a desired exchange probability of 20%. Each of the 48 replica simulations was run for 200 ns and exchanges between replica trajectories were attempted every 40 ps.

### 4.2. Structural Analysis

The whole trajectories of the classical MD simulations were used for structural analyses. However, regarding the REMD study, only the last 150 ns of the first ten trajectories generated at the lowest temperatures ranging from 290 to 313 K were analyzed (1.5 μs in total). Structural characterizations of the hIAPP dynamics were carried out using mainly the GROMACS tools, such as g_rms, g_gyrate or do_dssp [54]. The “gromos” method [55] implemented in g_cluster was used with a RMSD threshold of 0.2 nm to search for the most populated hIAPP conformations. The secondary structures propensity of the hIAPP residues were calculated using the STRIDE program [56,57]. These structural characteristics were grouped into three categories: helical (type H, G or I), extended (E or B) or coil (all other states).

## 5. Conclusions

In this study, we investigated the differences between the amidated and non-amidated hIAPP conformational ensembles, by performing extensive all-atom MD and REMD simulations. The secondary structure analyses clearly showed that the non-amidation of hIAPP induces a significantly lower propensity to form β-strands when compared to the amidated monomer. In contrast, both amidated and non-amidated peptides have a rather similar propensity to form helical structures at the N-terminal region. Since the fibrillization of the non-amidated hIAPP is significantly slower than that of the amidated peptide [22,23,24], our theoretical results support the hypothesis that the early steps of the peptide oligomerization in solution involve the association of β-hairpins or β-strand structures, and that its rate would be correlated to the lifetime of these transient conformations.

## Figures and Tables

**Table 1:** Structural characteristics of the 10 most populated clusters of the non-amidated hIAPP found by REMD simulations. The RMSD are calculated relative to the initial random coil conformation.

Cluster	Population (%)	RMSD (nm)	Gyration (nm)	α-Helix	β-Strand
01	4.6	1.00	0.88		
02	4.5	0.65	0.90	5–7, 16–21	
03	3.7	0.78	0.93		
04	3.6	0.30	0.89		
05	2.7	0.99	0.94		
06	2.3	0.88	0.97	5–7, 14–16	9–10, 34–35
07	2.1	0.87	0.86		
08	2.0	0.91	0.88		
09	1.9	0.75	0.89	5–11, 13–17	
10	1.9	0.90	0.90		

## Abbreviations

CINES	Centre Informatique National de l’Enseignement Supérieur (Montpellier, France)
GENCI	Grand Équipement National de Calcul Intensif (Paris, France)
GROMACS	GROningen MAchine for Chemical Simulations
IAPP	Islet Amyloid Polypeptide
HPC	High Performance Computer
LINCS	LINear Constraint Solver
MD	Molecular Dynamics
NPT	constant Number of particles Pressure and Temperature
OPLS-AA	Optimized Potentials for Liquid Simulations - All Atom
PME	Particle Mesh Ewald
REMD	Replica Exchange Molecular Dynamics
RMSD	Root Mean Square Deviation
SDS	Sodium Dodecyl Sulfate
SETTLE	ShakE and raTTLE
SPC/E	Simple Point Charge / Extended
STRIDE	STRucture IDEntification
